# Autophagy-related gene expression is an independent prognostic indicator of glioma

**DOI:** 10.18632/oncotarget.17719

**Published:** 2017-05-09

**Authors:** Huixue Zhang, Xiaoyan Lu, Ning Wang, Jianjian Wang, Yuze Cao, Tianfeng Wang, Xueling Zhou, Yang Jiao, Lei Yang, Xiaokun Wang, Lin Cong, Jianlong Li, Jie Li, He-Ping Ma, Yonghui Pan, Shangwei Ning, Lihua Wang

**Affiliations:** ^1^ Department of Neurology, The Second Affiliated Hospital, Harbin Medical University, Harbin, China; ^2^ Department of Neurology, Peking Union Medical College Hospital, Peking Union Medical College and Chinese Academy of Medical Sciences, Beijing, China; ^3^ Department of Neurosurgery, The Second Affiliated Hospital, Harbin Medical University, Harbin, China; ^4^ Department of Physiology, Emory University School of Medicine, Atlanta, GA, USA; ^5^ Department of Neurosurgery, The First Affiliated Hospital, Harbin Medical University, Harbin, China; ^6^ College of Bioinformatics Science and Technology, Harbin Medical University, Harbin, Heilongjiang, China

**Keywords:** autophagy, glioma, prognostic signature, survival

## Abstract

In this study, we identified 74 differentially expressed autophagy-related genes in glioma patients. Analysis using a Cox proportional hazard regression model showed that MAPK8IP1 and SH3GLB1, two autophagy-related genes, were associated with the prognostic signature for glioma. Glioma patients from the CGGA batches 1 and 2, GSE4412 and TCGA datasets could be divided into high- and low-risk groups with different survival times based on levels of MAPK8IP1 and SH3GLB1 expression. The autophagy-related signature was an independent predictor of survival outcomes in glioma patients. MAPK8IP1 overexpression and SH3GLB1 knockdown inhibited glioma cell proliferation, migration and invasion, and improved Temozolomide sensitivity. These findings suggest autophagy-related genes like MAPK8IP1 and SH3GLB1 could be potential therapeutic targets in glioma.

## INTRODUCTION

Autophagy is a dynamic process that degrades intracellular constituents in double membrane-bound vesicles (autophagosomes) during stress or nutrient deprivation [1]. Autophagosomes undergo stepwise initiation, nucleation, elongation, closure and degradation. Autophagy promotes metabolic homeostasis and could prevent degenerative diseases and cancers [2]. However, its role in tumorigenesis is controversial [3].

Recent studies have indicated that autophagy enables tumor cell survival or induces cell death depending on the cellular context [4]. Autophagy can suppress early stages of cancer development by eliminating damaged proteins and organelles, thereby mitigating cellular damage and limiting chromosomal instability [5, 6]. However, it can also promote tumor growth in low oxygen and nutrient conditions [7, 8]. Recent studies indicate that inhibition of autophagy suppresses tumor growth, promotes tumor cell death and overcomes therapy resistance [9]. In recent years, autophagy-related gene signature has robustly predicted clinical outcomes in pancreatic ductal adenocarcinoma and breast tumor [10, 11]. However, prognostic biomarkers for glioma based on autophagy have not been identified.

Malignant primary brain tumors are major causes of cancer-related death in children and young adults [12]. Glioblastoma (GBM) is the most frequent and lethal type of malignant glioma, which is highly infiltrative and rapidly progressive [13]. Despite surgery followed by standard radiotherapy and adjuvant chemotherapy, the prognosis of GBM remains poor with a median survival time of 12 to 15 months [14]. Although clinical and pathological subtype studies of glioma have increased in recent years, there is a need for prognostic markers and predictors of therapeutic response [15, 16]. Previous studies have reported association between autophagy and gliomas. Honokiol treatment increased autophagy markers, Beclin-1 and LC3-II in glioma cells [17]. Recently, pro-autophagy drugs were shown to inhibit tumor growth. Temozolomide (TMZ) was shown to increase survival time by inducing autophagy [18]. Temozolomide is approved for the standard treatment of newly diagnosed glioma in conjunction with radiotherapy [19]. Most studies investigating the role of autophagy in tumorigenesis have analyzed a limited number of autophagy-related genes in either cell lines or animal models. Hence, the prognostic value of global expression patterns of autophagy-related genes has not been realized in gliomas.

Since large-scale expression data is available, it is feasible to study if the global gene expression pattern of autophagy-related genes can predict clinical outcomes of glioma patients. The Human Autophagy Database (HADb) integrates all genes that are directly or indirectly involved in autophagy based on PubMed and other biological public databases [20]. In this study, we analyzed the differential expression of autophagy-related genes in glioma samples to identify the enriched pathways and their biological functions. We also investigated if autophagy related genes are associated with glioma prognosis. We identified two genes, MAPK8IP1 and SH3GLB1 that accurately predicted the clinical outcome of glioma patients. SH3GLB1 or Bif-1 was shown previously to be involved in the formation of autophagosomes [21]. Also, MAPK8IP1 or JIP1 was shown to regulate trafficking of autophagosomes in neurons [22]. Therefore, we explored the mechanism of autophagy regulation by MAPK8IP1 and SH3GLB1 [23]. Furthermore, we investigated the role of MAPK8IP1 and SH3GLB1 on glioma cell proliferation, migration, invasion and their effect on Temozolomide treatment. Overall, our data suggested that autophagy-related genes play important roles in cancer and are potential prognostic markers and targets for glioma.

## RESULTS

### Identification of differentially expressed autophagy genes in gliomas

**Figure 1:**
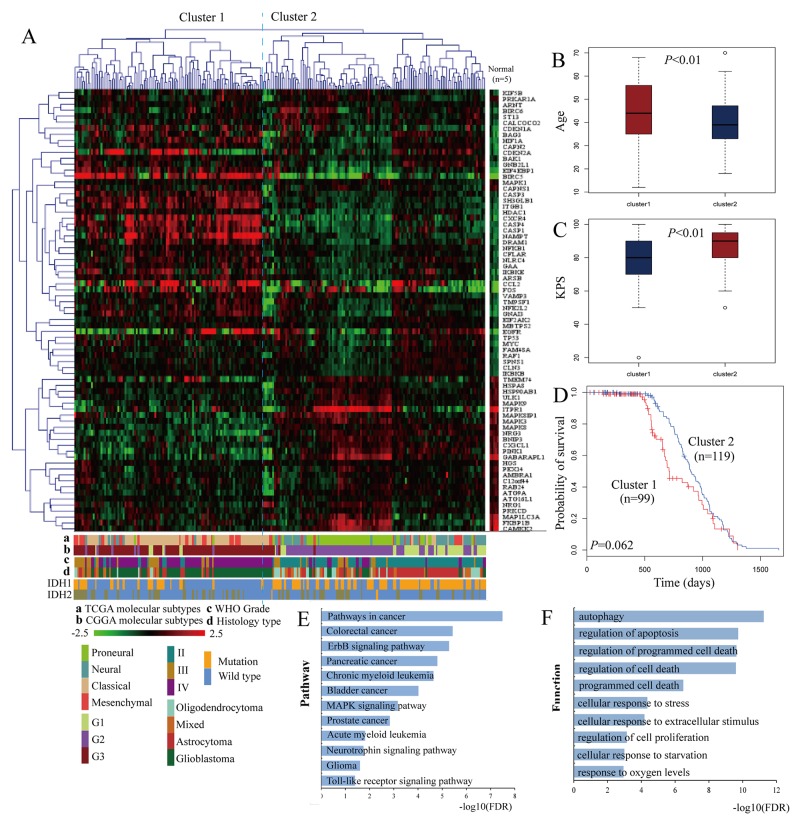
Hierarchical clustering analysis of the differentially expressed autophagy genes **(A)** The glioma samples are divided into two clusters. The corresponding data tracks highlight different clinical and molecular features associated with the two clusters like WHO grades, TCGA or CGGA molecular subtypes, histology subtypes and IDH1 or IDH2 mutations. **(B)** Age **(C)** KPS and **(D)** Overall survival of patients in the two clusters. **(E)** Significant pathways enriched by the differentially expressed autophagy genes. **(F)** Significant biological functions enriched by the differentially expressed autophagy genes.

The flowchart of our methodology is shown in Supplementary Figure 1. The CGGA batch 1 dataset was selected to identify differentially expressed autophagy genes in gliomas. Among the 234 autophagy-related genes, 74 were differentially expressed in the glioma samples (*P*<0.05; Figure 1A). The CGGA batch 1 glioma samples were divided into clusters 1 and 2 with distinct clinical outcomes (Figure 1A). The tumors of cluster 1 were highly malignant and belonged to high WHO grade, classical and mesenchymal subtypes, G3 subgroup, glioblastoma and harbored wild type isocitrate dehydrogenase 1 (IDH1). The tumors of cluster 2 belonged to low WHO grade with IDH1 mutation and indicated better survival. Yan *et al.* had previously demonstrated that patients with IDH1 or IDH2 mutations had better outcomes than those with wild-type IDH1 or IDH2 [24]. The patients of cluster 1 were older with lower Karnofsky Performance Status (KPS) than patients of cluster 2 (Figure 1B–1C). The patients of cluster 1 also showed poor overall survival than cluster 2 patients (HR=0.70, 95% CI=0.49-1.02, *P*=0.062; Figure 1D).

### Pathways and biological functions of differentially expressed autophagy genes in gliomas

We performed functional enrichment analysis with DAVID to identify risk pathways and biological functions associated with the differentially expressed autophagy genes. As shown in Figure 1E, we identified 15 KEGG pathways with a FDR<0.05 such as ErbB signaling, MAPK signaling and neurotrophin signaling related to glioma. The mitogen-activated protein kinase (MAPK) signaling pathway is essential for migration and invasion of glioma [25]. Drugs like Oleanolic Acid decrease the metastatic ability of glioma cells by suppressing the MAPK signaling [26]. We also found that the biological functions of the differentially expressed autophagy genes were related to autophagy, apoptosis, and cellular responses to stress, starvation and extracellular stimuli (Figure 1F). Collectively, autophagy played an important role in the pathogenesis of glioma.

### Autophagy-related signature predicts survival in glioma

**Figure 2:**
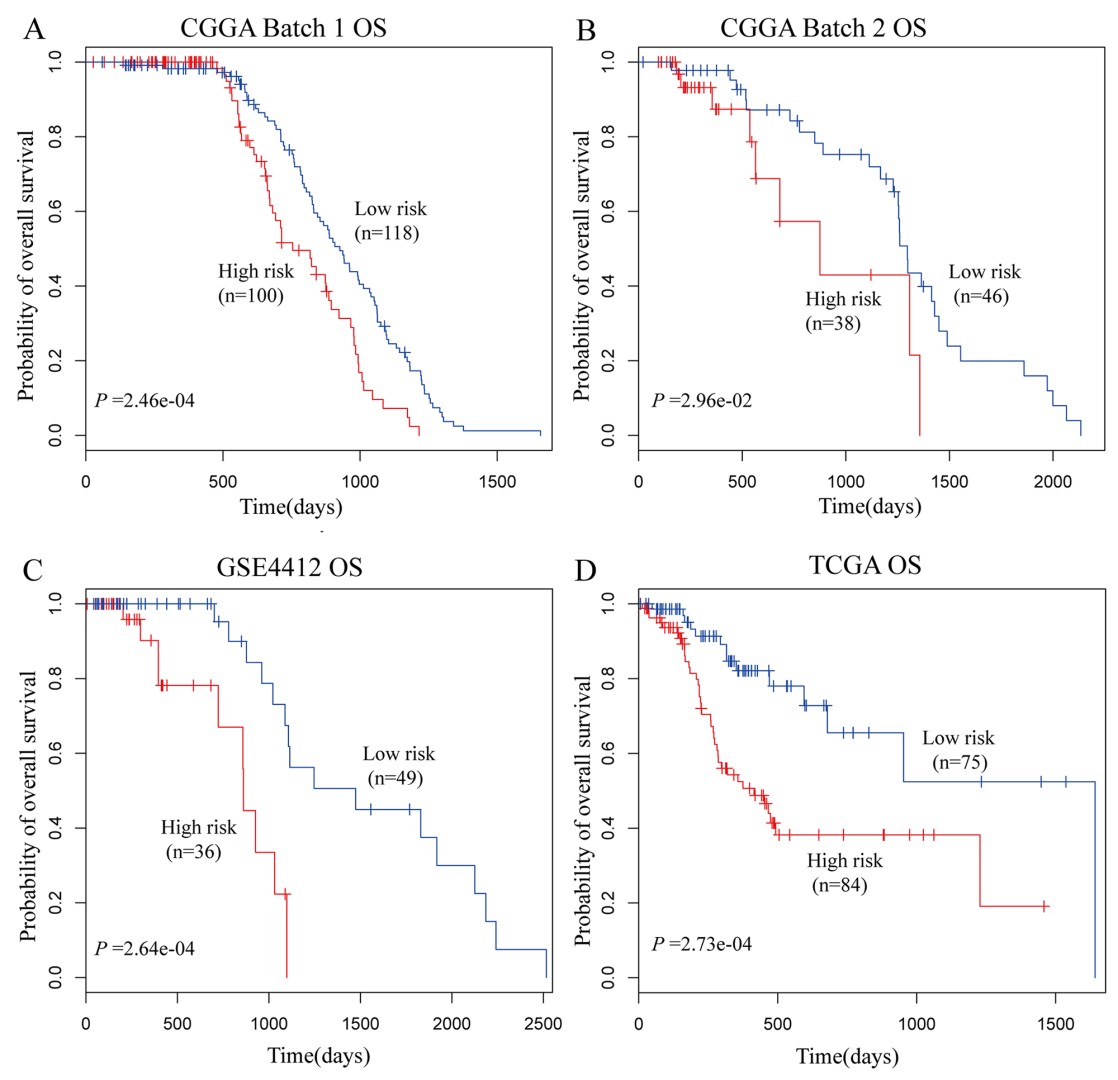
Overall Survival of glioma patients based on autophagy-related signature Overall survival of **(A)** 218 glioma patients in the training data set; **(B)** 84 glioma patients in the test data set; **(C)** 85 glioma patients in the GSE4412 cohort; and **(D)** 159 glioma patients in the TCGA cohort.

To investigate if the differentially expressed autophagy genes are prognostic biomarkers in glioma, we assigned the CGGA batch 1 dataset as the training set. Based on median survival time, the patients were divided into good and poor prognosis groups. Among the 74 autophagy genes, 26 were differentially expressed between these two groups. We then used Cox proportional hazard regression analysis to select the autophagy genes associated with glioma survival time (*P*<0.05). Our analysis showed that MAPK8IP1 and SH3GLB1 were significantly associated with overall survival and used them to construct a signature by generating a risk score for each patient in the training set. Then, the patients were divided into high-risk (n=100) or low-risk (n=118) groups using the median risk score (0.05) as the cutoff point. The clinical characteristics of the 218 patients in the training set are shown in Supplementary Table 1. Our data showed that patients with high-risk scores had shorter median survival time than patients with low-risk scores (HR=0.50, 95% CI= 0.35-0.75, *P* =2.46×10^-4^; Figure 2A).

To validate the autophagy-related signature, we selected the CGGA batch 2 as the testing dataset and calculated the autophagy-related signature risk score for each of the 84 patients. The patients were classified into high-risk or low-risk groups using the median risk score as the cutoff. The clinical characteristics of the 84 patients are shown in Supplementary Table 1. Similar to the findings from the CGGA batch 1 training set, patients from CGGA batch 2 with high-risk autophagy-related signature had shorter median overall survival than patients with low-risk autophagy-related signature (HR=0.41, 95% CI= 0.18-0.94, *P* =2.96×10^-2^; Figure 2B).

We further chose two independent glioma datasets GSE4412 and TCGA for analysis. Supplementary Table 1 shows the clinical characteristics of the patients in these two independent cohorts. Patients were classified as high-risk or low-risk groups based on their autophagy-related signature risk scores. In the GSE4412 cohort, patients with high-risk scores had shorter overall survival than patients with low-risk scores (HR=0.17, 95% CI= 0.06-0.50, *P* =2.64×10^-4^; Figure 2C). Likewise, patients with high-risk scores in the TCGA dataset had shorter overall survival than patients with low-risk scores (HR=0.33, 95% CI= 0.17-0.62, *P* =2.73×10^-4^; Figure 2D).

**Figure 3:**
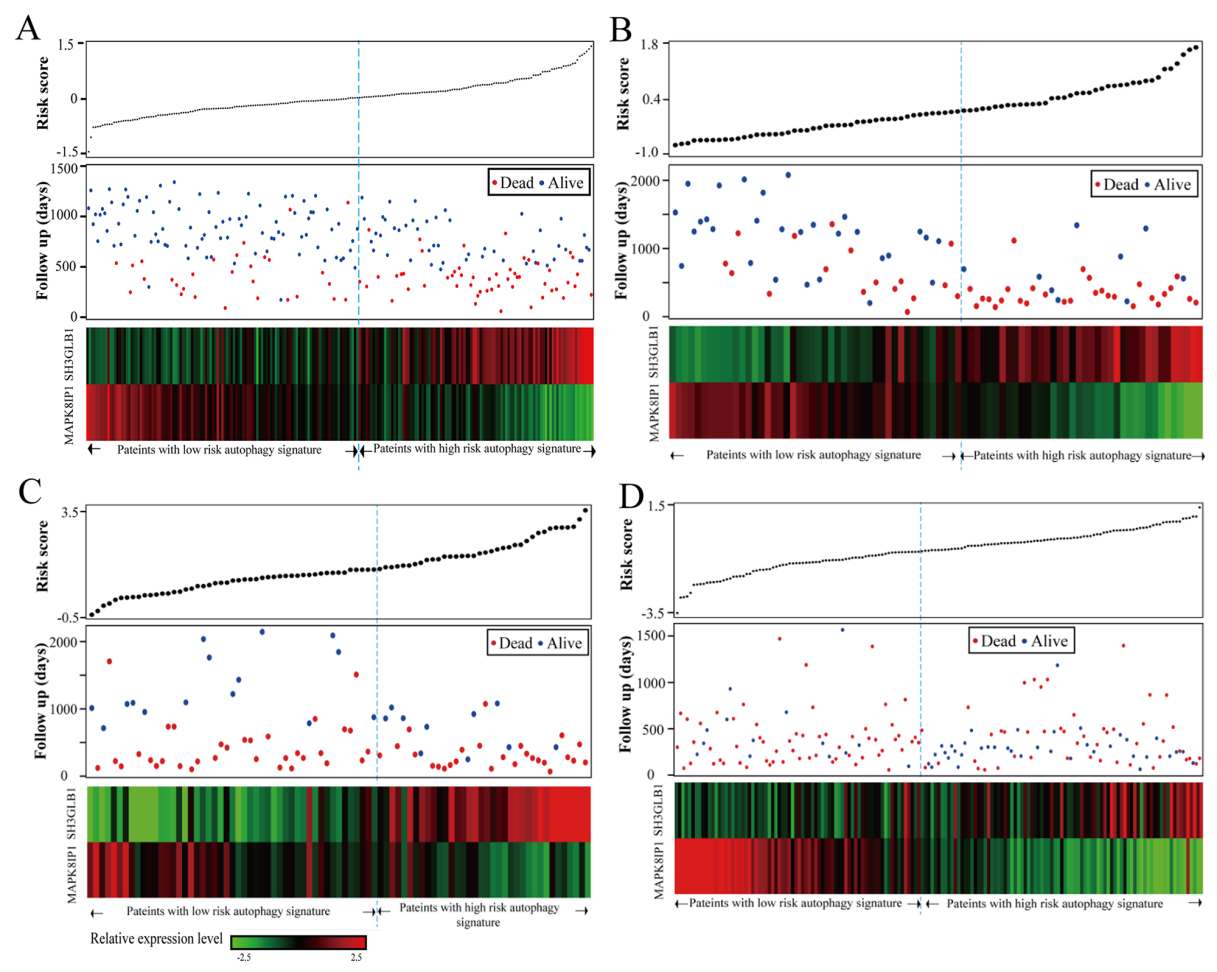
Autophagy related risk score analysis of glioma patients in the four datasets Autophagy related risk score analysis in **(A)** CGGA Batch 1, **(B)** CGGA batch 2, **(C)** GSE4412 and **(D)** TCGA. The autophagy related risk score distribution is shown in the upper panel. The patient survival status is shown in the middle panel. The color coded autophagy related gene expression proﬁles of glioma patients is shown in the bottom panel. The rows represent autophagy related genes and columns represent patients. The light blue dotted line represents the median cutoff point dividing patients into low- and high-risk groups.

Figure 3A shows comparison of MAPK8IP1 and SH3GLB1 expression with risk scores and survival status of CGGA batch 1 patients. We observed that gliomas with high risk scores expressed SH3GLB1, whereas those with low risk scores expressed MAPK8IP1. Similar results were obtained for CGGA batch 2 (Figure 3B), GSE4412 (Figure 3C) and TCGA (Figure 3D) datasets.

### Autophagy-related signature is an independent predictor of glioma

We conducted multivariate Cox regression analysis to compare the predictive power of the autophagy-related signature with other clinical factors such as age, gender, stage, KPS, IDH1 or IDH2 mutation. In CGGA batch 2 dataset, univariate analysis revealed that the autophagy-related signature was significantly associated with overall survival (*P*=0.03) and marginally insignificant in the multivariate analysis (*P*=0.056). The other clinical factors like age, gender, histology, WHO grade, IDH1 and IDH2 mutations were not significant (Table 1). Both univariate and multivariate analyses of the TCGA cohort revealed that the autophagy-related signature was associated with overall survival (*P*=2.73×10^-4^ and *P=*0.024, respectively), whereas factors like age, gender and KPS were not significant (Table 1). In the GSE4412 cohort, univariate analysis showed that the autophagy-related signature was associated with overall survival (*P*=2.64×10^-4^) and marginally insignificant in the multivariate analysis (*P*=0.052; Table 1). Collectively, our analysis demonstrated that the autophagy-related signature independently predicted glioma patient survival.

**Table 1:** Univariate and multivariate Cox regression analysis of overall survival in three datasets

Characteristics	Univariate analysis		Multivariate analysis	
	HR(95% CI)	*P* value	HR (95% CI)	*P* value
**CGGA batch 2 (n=84)**				
Autophagy signature	0.41 (0.18,0.94)	2.96×10^-2^	0.31 (0.10,1.03)	0.056
Age at diagnosis	1.01 (0.98,1.05)	0.496	0.99 (0.94,1.04)	0.654
Gender	1.24 (0.62,2.47)	0.543	0.73 (0.31,1.74)	0.480
Histology	1.00 (0.72,1.40)	0.986	0.99 (0,55,1.78)	0.964
WHO Grade	1.37 (0.86,2.19)	0.187	0.91 (0.34,2.46)	0.848
IDH1 mutation	1.05 (0.53,2.08)	0.887	1.25 (0.44,3.57)	0.671
IDH2 mutation	0.38 (0.05,2.85)	0.326	0.32 (0.03,3.90)	0.375
**TCGA (n=159)**				
Autophagy signature	0.33 (0.17,0.62)	2.73×10^-4^	0.46 (0.23,0.90)	0.024
Age at diagnosis	1.01 (0.98,1.03)	0.628	0.99 (0.96,1.01)	0.287
Gender	0.92 (0.51,1.67)	0.795	0.72 (0.35,1.45)	0.355
KPS	0.99 (0.97,1.02)	0.634	0.98 (0.96,1.01)	0.239
**GSE4412 (n=85)**				
Autophagy signature	0.17 (0.06,0.50)	2.64×10^-4^	0.28 (0.07,1.01)	0.052
Age at diagnosis	1.09 (1.04,1.14)	4.83×10^-4^	1.07 (1.01,1.13)	0.017
Gender	2.60 (1.01,6.74)	0.049	1.72 (0.55,5.34)	0.35
Histology	0.59 (0.39,0.90)	0.013	0.95 (0.50,1.85)	0.91
WHO Grade	5.11 (1.81,14.45)	0.002	2.46 (0.33,18.04)	0.38

### Roles of MAPK8IP1 and SH3GLB1 in autophagy regulation and glioma

**Figure 4:**
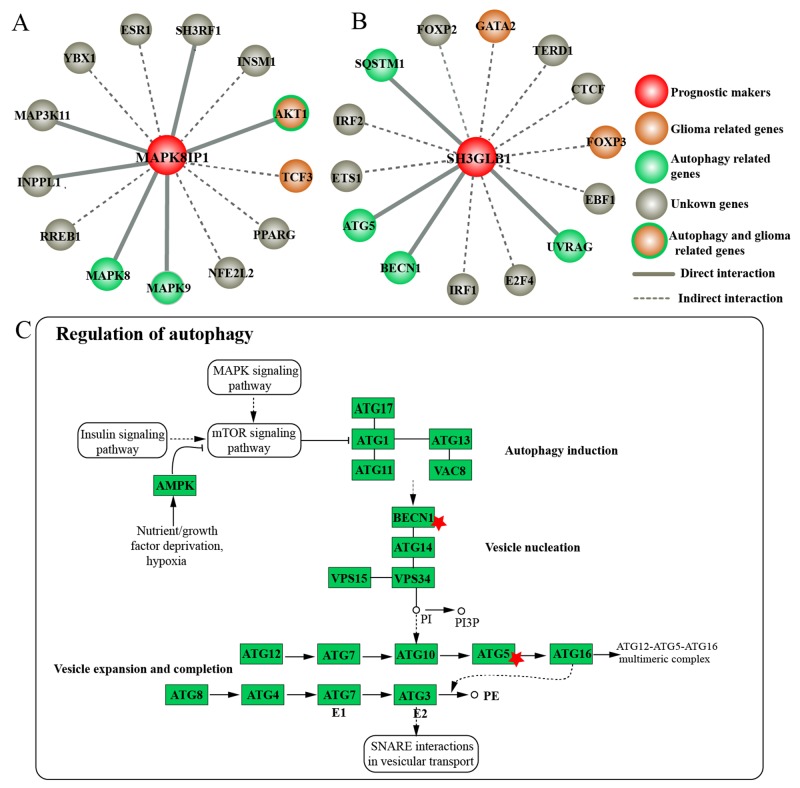
Roles ofMAPK8IP1 and SH3GLB1 in autophagy regulation The first interacting neighbors of **(A)** MAPK8IP1 and **(B)** SH3GLB1 extracted from Autophagy Regulatory Network. Solid line represents direct interaction while dashed line shows indirect interaction. Green, orange, red and grey circles represent autophagy-related genes, glioma-related genes, our markers and unknown genes, respectively. **(C)** Regulation of autophagy pathway downloaded from the KEGG database. Red star indicates genes interacting with SH3GLB1.

Both MAPK8IP1 and SH3GLB1 are directly related to autophagy [21, 22]. To elucidate the role of MAPK8IP1 and SH3GLB1 in autophagy regulation, we extracted their first interacting neighbors from the Autophagy Regulatory Network (ARN, http://autophagy-regulation.org). ARN is a comprehensive database that contains manually curated and predicted interactions of autophagy components [23]. ARN also connects autophagy components and their signaling pathways from the SignaLink2 resource [27]. We found that MAPK8IP1 directly interacted with three autophagy related proteins AKT1, MAPK8 and MAPK9 (Figure 4A). Akt1 inhibited autophagy by down-regulating UVRAG [28]. MAPK8 or JNK1 activated autophagy and mediated starvation-induced Bcl-2 phosphorylation [29]. MAPK9 or JNK2 also induced autophagy [30]. SH3GLB1 directly interacted with four autophagy-related proteins, ATG5, BECN1, SQSTM1 and UVRAG (Figure 4B). The autophagy adaptor protein SQSTM1 and ATG5 mediated autophagosome formation [31]. BECN1 or Beclin 1 is an autophagosome initiator that is overexpressed in glioma cells, but not expressed in normal brain neurons or glial cells [32]. UVRAG, also known as VPS38 and p63, interacts with BECN1 leading to activation of autophagy and inhibition of tumorigenesis [33]. These results showed that both MAPK8IP1 and SH3GLB1 interacted with other autophagy-related components.

Furthermore, we downloaded the regulation of autophagy pathway from the KEGG database (Figure 4C). We observed that BECN1 participated in vesicle nucleation whereas ATG5 played a role in vesicle expansion and completion of autophagy. Also, SH3GLB1 directly interacted with BECN1 and ATG5. Also, transcription factors interacting with MAPK8IP1 and SH3GLB1 were involved in multiple pathways like TGF, Hedgehog and WNT/Wingless signaling, which were associated with glioma [34, 35]. These results indicated that MAPK8IP1 and SH3GLB1 not only interacted with other autophagy-related proteins, but were also involved in multiple pathways related to glioma.

### MAPK8IP1 overexpression inhibits proliferation, migration and invasion of glioma cells and increases sensitivity to Temozolomide

MAPK8IP1 was down-regulated in glioma samples from the CGGA Batch 1 dataset (Supplementary Figure 2A). Therefore, we analyzed the expression of MAPK8IP1 in five glioma cell lines by qRT-PCR. Compared to oligodendrocytes, three glioma cell lines (SNB19, U251, T98G) had low MAPK8IP1 mRNA levels, whereas two glioma cell lines (LN229, U87) showed higher MAPK8IP1 transcripts (Supplementary Figure 3A). Subsequently, we selected the T98G glioma cell line for further experiments.

**Figure 5:**
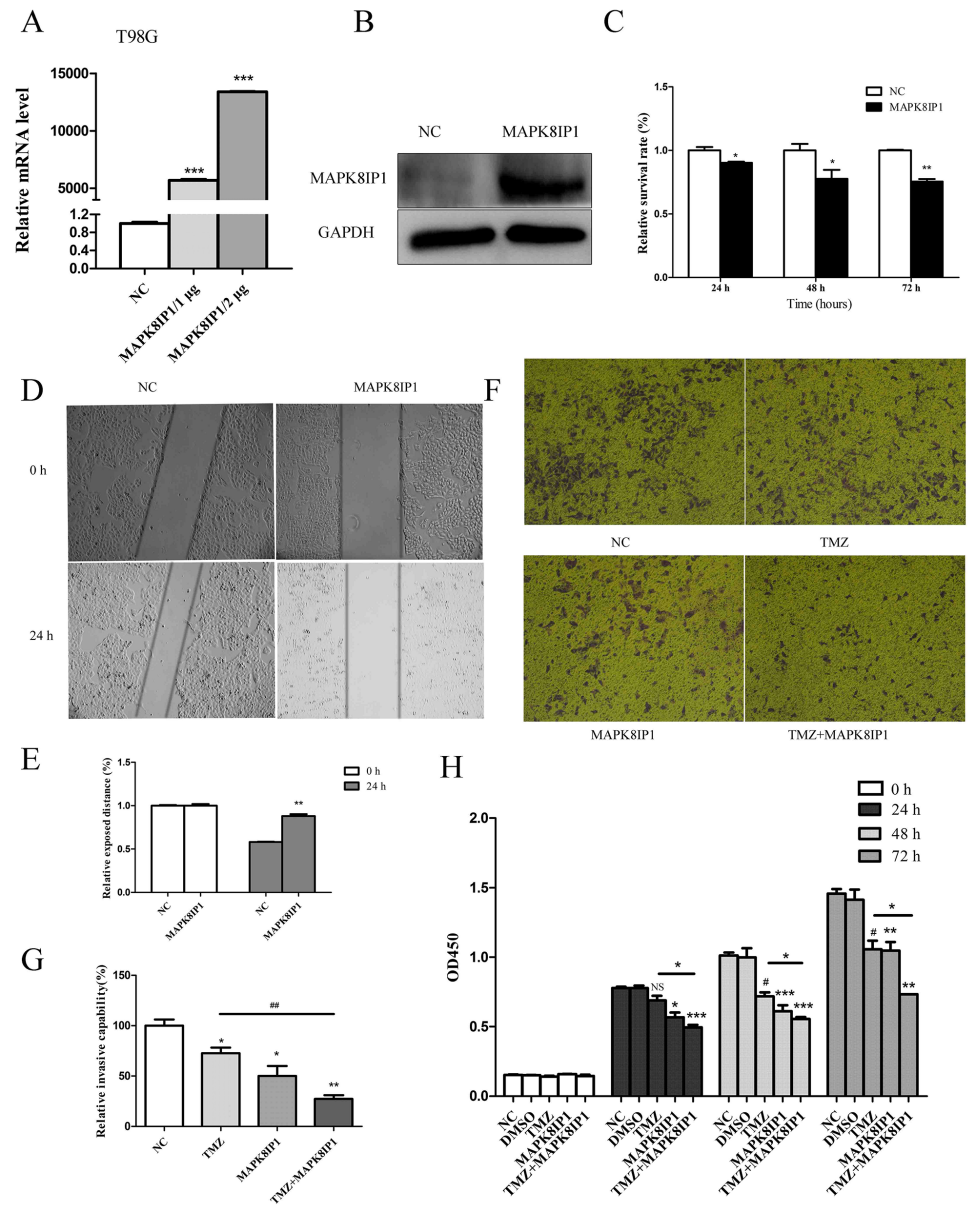
MAPK8IP1 overexpression inhibits proliferation, migration, invasion of glioma cells and improves sensitivity to Temozolomide **(A)** MAPK8IP1 mRNA and **(B)** MAPK8IP1 protein levels increased upon transfection with the MAPK8IP1 plasmid. **(C)** CCK-8 assay shows cell proliferation after transfection with MAPK8IP1 at different time points. **(D)** Representative images (50x) of wound healing assay showing the scratch photographed at 0 and 24h after transfection. **(E)** The relative exposed area between cells in the scratch area are shown for the MAPK8IP1 overexpressing cells compared to control. **(F)** Representative images (50x) of transwell assays of T98G cells after MAPK8IP1transfection with or without Temozolomide treatment are shown. **(G)** Histogram showing number of invading MAPK8IP1 overexpressing T98G cells with or without TMZ treatment. **(H)** CCK-8 assay showing proliferation of MAPK8IP1 overexpressing T98G cells with or without Temozolomide treatment at 24, 48 and 72 h. Data represent mean ± SEM of three replicates. ^*^P<0.05; ^**^P<0.01; ^***^ *P*<0.001.

To investigate the effect of MAPK8IP1 on the biological behavior of glioma cells, we transfected T98G cells with the MAPK8IP1 plasmid and demonstrated that MAPK8IP1 was overexpressed by qRT-PCR and western blot analysis (Figure 5A, 5B). Then, we performed CCK-8 assay to examine the effect of MAPK8IP1 overexpression on proliferation and observed that cell proliferation was inhibited at 24, 48 and 72h (*P*<0.05; Figure 5C). Since the malignant glioma cell lines show highly invasive growth characteristics, both *in vitro* and *in vivo*, we performed wound-healing and transwell invasion assays to test the effects of MAPK8IP1 overexpression. We observed that MAPK8IP1 overexpression inhibited migration and invasion of T98G cells compared to controls (Figure 5D-5G). Then, we explored the effects of MAPK8IP1 overexpression on TMZ therapy by performing CCK-8 and transwell invasion assays. Treatment of T98G cells that overexpressed MAPK8IP1 with 100μM Temozolomide [36] suppressed proliferation and invasion suggesting that MAPK8IP1 overexpression increased the sensitivity of gliomas to Temozolomide treatment (Figure 5H).

### SH3GLB1 downregulation inhibits proliferation, migration and invasion of glioma cells and increases sensitivity to Temozolomide

We observed that SH3GLB1 was up-regulated in glioma samples from the CGGA Batch 1 dataset (Supplementary Figure 2B). We analyzed expression of SH3GLB1 in five glioma cell lines using qRT-PCR and observed that SH3GLB1 was upregulated in LN229 glioma cells compared to oligodendrocytes (Supplementary Figure 3B). Therefore, we investigated the effect of SH3GLB1 downregulation in LN229 glioma cells.

**Figure 6:**
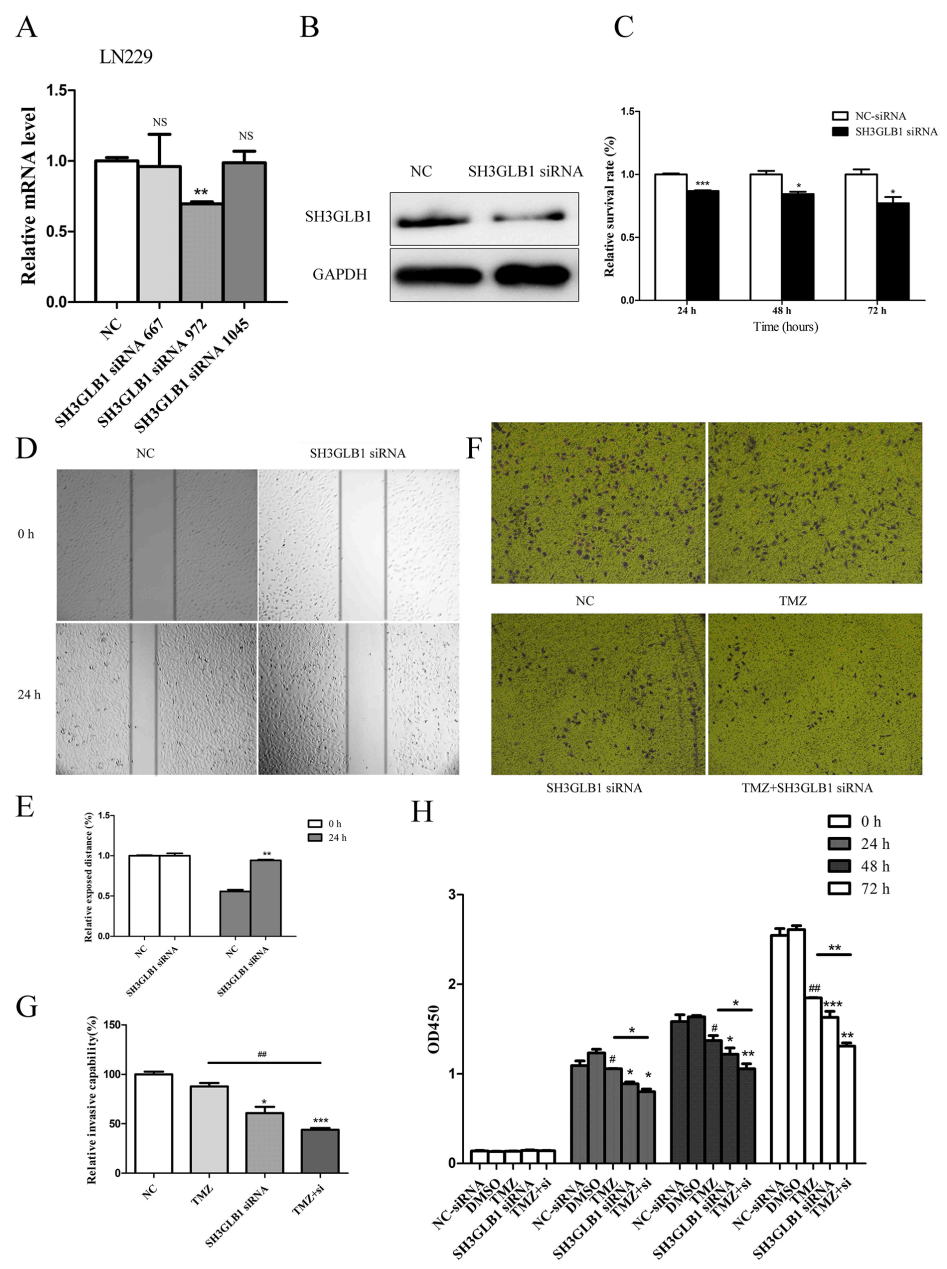
SH3GLB1 downregulation inhibits proliferation, migration and invasion of glioma cells and improves sensitivity to Temozolomide **(A)** QRT-PCR data showing the knockdown efficiency of 3 siRNAs against SH3GLB1. The relative SH3GLB1mRNA levels upon transfection with SH3GLB1-siRNA-972. **(B)** The relative SH3GLB1protein levels after SH3GLB1 siRNA knockdown. **(C)** CCK8 assay showing cell proliferation at different time points after SH3GLB1 knockdown. **(D)** Representative images (50x) of wound healing assay showing the scratch photographed at 0 and 24h after LN229 cells were transfected with SH3GLB1 siRNA. **(E)** The relative exposed area between cells in the scratch area for transfected cells normalized against control. **(F)** Representative images (50x) of transwell assays of LN229 cell line after SH3GLB1 siRNA knockdown with or without Temozolomide. **(G)** Histogram showing number of invading SH3GLB1 siRNA knockdown LN229 cells with or without Temozolomide. **(H)** CCK-8 assay showing proliferation of SH3GLB1 siRNA knockdown LN229 cells with or without Temozolomide at 24, 48 and 72h. Data represent mean ± SEM of three replicates. ^*^P < 0.05; ^**^P < 0.01; ^***^ *P*< 0.001.

We transfected three SH3GLB1 siRNAs in LN229 glioma cells and analyzed SH3GLB1 mRNA levels by qRT-PCR. We observed that only siRNA-972 knocked down SH3GLB1 significantly (Figure 6A). Western blot analysis confirmed that siRNA-972 downregulated SH3GLB1 protein (Figure 6B). Then, we examined the effects of SH3GLB1 downregulation on proliferation of LN229 glioma cells by CCK-8 assay. We observed reduced growth and cell proliferation upon SH3GLB1 downregulation in LN229 cells at 24, 48 and 72h (Figure 6C; *P*< 0.05). Then, we performed wound-healing and transwell invasion assays to test the effects of SH3GLB1 downregulation on cell migration and invasion. We observed that migration and invasion of LN229 cells was inhibited by SH3GLB1 downregulation (Figure 6D-6G). We further explored the effects of downregulating SH3GLB1 on TMZ therapy by performing CCK-8 and transwell invasion assays. We observed that treating SH3GLB1 downregulated LN229 glioma cells with 100μM TMZ suppressed glioma cell proliferation and invasion. This suggested that SH3GLB1 downregulation increased the sensitivity of glioma cells to Temozolomide (Figure 6H).

## DISCUSSION

Glioma is a progressive disease that requires effective prognostic markers for diagnosis and treatment. Recently, effective computational models have been constructed to identify disease-related mRNA or non-coding RNA biomarkers [37-41]. There is increasing evidence that autophagy plays a significant role in tumorigenesis and cancer therapy. However, most studies have focused on autophagy at a single gene, protein or miRNA level using cell lines or animal models. In our study, we used high-throughput expression data to investigate the differential expression profiling of autophagy-related genes in glioma. Hierarchical clustering analysis revealed that the glioma samples could be classified into 2 groups based on autophagy-related genes with different survival times, histology types and stages and age groups.

Functional analysis revealed that most of the pathways enriched by the differentially expressed autophagy-related genes were cancer related suggesting that dysregulation of autophagy played a role in initiation and progression of glioma. We also identified two genes, MAPK8IP1 and SH3GLB1, which precisely predicted clinical outcomes of glioma patients. We validated our findings with 3 independent cohorts of glioma patients. SH3GLB1 forms a complex with Beclin 1 through UVRAG to enhance PI3KC3 lipid kinase activity and induces autophagosome formation during nutrient starvation [21]. Further, Fu *et al.* demonstrated that MAPK8IP1 regulated trafficking of autophagosomes [22]. Our data suggested that both SH3GLB1 and MAPK8IP1 interacted with other autophagy-related components and were involved in the pathogenesis of glioma. However, their role in glioma had not been investigated. We demonstrated that MAPK8IP1 overexpression and SH3GLB1 knockdown inhibited the proliferation, migration and invasion of glioma cells and increased sensitivity to Temozolomide. This suggested that MAPK8IP1 played a tumor suppressor role, whereas SH3GLB1 was a oncogene in glioma. In a previous study, MAPK8IP1 was shown to interact with JNKs, MKK7, and members of the mixed lineage kinases (MLKs) to regulate the JNK signaling pathway [42]. Tawadros *et al.* demonstrated that upregulation of MAPK8IP1 prevented activation of JNK signaling pathway [43]. Zhou *et al.* also reported that inhibition of JNK signal pathway suppressed migration and invasion of glioma cells [44].

Autophagy is a dynamic process that responds to stress or starvation by degrading damaged proteins and cytoplasmic organelles. However, the role of autophagy in cancer is unclear. In our study, upregulation of the autophagy-related gene MAPK8IP1 suppressed proliferation, migration and invasion of glioma cells. Concurrently, down-regulation of another autophagy-related gene SH3GLB1 also inhibited proliferation, migration and invasion of glioma cells. This suggested that the two autophagy related genes, SH3GLB1 and MAPK8IP1 had opposite effects on glioma progression.

Further, autophagy has been identified as a novel therapeutic target for cancers. Sui *et al.* reported that modulation of autophagy improved response to chemoradiotherapy [9]. In recent years, drugs like Chloroquine and RAD001 have targeted autophagy to increase sensitivity of glioma to Temozolomide treatment [45, 46]. Temozolomide is approved for the standard treatment of newly diagnosed glioma patient and shown to improve prognosis [19]. In our study, we investigated the effects of two autophagy-related genes, MAPK8IP1 and SH3GLB1, on Temozolomide efficacy. We demonstrated that MAPK8IP1 overexpression and SH3GLB1 downregulation increased the sensitivity of glioma cells to Temozolomide treatment.

In summary, we demonstrated that autophagy-related gene expression patterns are independent predictors of overall survival of glioma patients. We also showed that MAPK8IP1 overexpression and SH3GLB1 knockdown inhibited proliferation, migration and invasion of glioma cells in addition to improving sensitivity to TMZ treatment. In conclusion, our study demonstrated that targeting autophagy-related genes is a promising future therapeutic strategy for gliomas.

## MATERIALS AND METHODS

### Human autophagy related genes

The 234 autophagy-related genes were obtained from the Human Autophagy Database (HADb, http://autophagy.lu/clustering/index.html). This database contains genes directly or indirectly involved in the autophagy process based on literature in PubMed and biological public databases [20].

### Human Glioma expression datasets

#### The Chinese Glioma Genome Atlas datasets

The mRNA microarray of glioma samples and their clinical information was obtained from the Chinese Glioma Genome Atlas (CGGA, http://www.cgga.org.cn/) [47]. The mRNA microarray of CGGA, produced by Agilent Whole Human Genome Array was divided into two batches. CGGA Batch 1 contained 5 normal samples and 220 diffuse glioma samples including 97 WHO grade II (58 astrocytomas, 22 oligoastrocytomas and 17 oligodendroglioma), 34 grade III (8 anaplastic astrocytomas, 11 anaplastic oligodendroglioma and 15 anaplastic oligoastrocytomas) and 89 grade IV (85 primary and 4 secondary GBMs) patients. Two glioma samples that lacked survival information were excluded for survival analysis. CGGA Batch 2 contained 85 diffuse glioma samples including 29 WHO grade II (11 astrocytomas, 8 oligoastrocytomas and 10 oligodendroglioma), 17 grade III (7 anaplastic astrocytomas, 3 anaplastic oligodendroglioma and 7 anaplastic oligoastrocytomas) and 39 GBM (35 primary and 4 secondary GBMs) patients. One glioma sample that lacked survival information was excluded for further analysis. The expression profiles of these two batches had already been normalized.

#### Gene Expression Omnibus dataset

One independent glioma cohortproduced by HGU133A platform was extracted from Gene Expression Omnibus (GEO) database (accession number: GSE4412) [48]. The *series_matrix.txt* format of this dataset was downloaded and all probes were mapped based on their EnterzGeneID. When multiple probes mapped to the same gene ID, the mean expression value of the gene was used. The GSE4412 dataset contained 85 glioma samples including 26 WHO grade III and 59 WHO grade IV GBM patients.

#### The Cancer Genome Atlas dataset

Another independent gene expression dataset produced by Illumina-HiSeq platform was downloaded from TCGA database (http://tcga-data.nci.nih.gov/docs/publications/gbm_exp/). The data was quantile normalized and background corrected. The average expression values were calculated for duplicated samples. Then, the expression values of autophagy genes were extracted from the normalized gene expression profiles. After excluding samples without survival information, 159 glioma samples were analyzed.

### Differential autophagy gene expression analysis

We selected the CGGA batch 1 dataset with glioma and normal samples to investigate the global autophagy gene expression patterns. A two-tailed t-test (*P*<0.05) was used to identify differentially expressed autophagy genes in glioma samples. Then, the differentially expressed autophagy genes were analyzed by hierarchical clustering. The pathways and biological functions of the differential autophagy genes were identified by enrichment analysis using DAVID Bioinformatics Resources [49].

### Glioma patient survival analysis

The CGGA Batch 1 dataset was selected as the training set to analyze prognostic signatures and the disease samples were divided into good and poor prognosis groups based on the median survival time. The differentially expressed autophagy-related genes in these two groups were identified based on the Student’s t-test (*P*<0.05). Then, Cox proportional hazard analysis was performed to identify the differentially expressed autophagy-related genes that correlated with overall survival (*P*<0.05). Each patient was assigned a risk score based on the expression level of the autophagy-related genes and the Cox regression coefficient [Risk score = -0.441×(expression value of MAPK8IP1) + 0.311×(expression value of SH3GLB1)]. Patients with high risk scores had poor clinical outcomes.

The patients in the training data set were divided into high- and low-risk groups using the median autophagy-related signature risk score as the cut-off point. The differences in patient characteristics for the high- and low-risk groups were analyzed by Student’s t-test for continuous variables or Fisher’s exact test for categorical variables. The Kaplan-Meier method was used to estimate overall survival. The differences in survival between high- and the low-risk patients was analyzed by two-sided log-rank test. The performance of this autophagy-related signature risk score model was validated using CGGA batch 2, GSE4412 and TCGA cohorts of glioma patients.

The multivariate Cox regression analysis was used to investigate if the autophagy-related signature was an independent predictor of overall survival in comparison to cancer stages, histology, Karnofsky Performance Status (KPS) and isocitrate dehydrogenase 1 (IDH1) or IDH2 mutations in the CGGA batch 2, GSE4412 and TCGA cohorts of glioma patients. The autophagy-related signature, age, gender, cancer stages, and histology subtypes were used as covariates. *P* value < 0.05 was considered significant.

### Glioma cell culture

Human glioma cell lines SNB19, U251, T98G, LN229 and U87 were obtained from the Chinese Academy of Sciences Cell Bank, Shanghai. The human oligodendroglial cell line (Olig) was a kind gift from Prof. Xia Li of Harbin Medical University. The cells were maintained in Dulbecco’s modified Eagle’s medium supplemented with 10% fetal bovine serum (Biological Industries, Israel) at 37°C and 5% CO2.

### MAPK8IP1overexpressing plasmid, SH3GLB1small interfering RNAs and TMZ treatment

The MAPK8IP1 (NM_005456) plasmid and the SH3GLB1 and control siRNAs were purchased from GenePharma (Shanghai, China). The sequences of the 3 SH3GLB1 and the control siRNAs are listed in Supplementary Table 2. A stock solution of 50mM TMZ (Sigma-Aldrich, USA) was prepared in DMSO (Sigma).

### RNA extraction, reverse transcription (RT) and quantitative real-time PCR analysis

Total RNA was extracted using TRIzol (Takara). Quantitative real-time PCR (qRT-PCR) assays were performed to measure the expression level of MAPK8IP1 and SH3GLB1 using the FastStart Universal SYBR Green Master (Roche Diagnostics) according to the manufacturer’s instructions. GAPDH was used to normalize MAPK8IP1 and SH3GLB1 expression. Real-time PCR was performed in triplicate in a LightCycler96 (Roche Diagnostics) and the data was analyzed by the Ct method [50]. The following gene specific primers were used: SH3GLB1 (F: CACCTCTCCT-TCCAACCTCA; R: CCATTCCAACAACACTGAACA; Sangon Biotech); MAPK8IP1 (F: CACCACGCTCA-ACCTCTTTC; R: GTCTGCTCCCCTGTCTTCAG; Sangon Biotech); GAPDH (forward, 5′-TGGACTCCAC-GACGTACTCAG-3′; GAPDH reverse, 5′-CGGGA-AGCTTGTCATCAATGGAA-3′; Invitrogen).

### Western Blot analysis

Western Blot (WB) analysis was performed as previously described [51]. Mouse anti-GAPDH (1:1000, ZSGB-BIO), Rabbit anti-MAPK8IP1 (1:200, Proteintech), Rabbit anti-SH3GLB1 (1:500, Proteintech), and HRP-labeled secondary antibody (1:3000, ZSGB-BIO) were used to quantify MAPK8IP1, SH3GLB1 and GAPDH (control) levels.

### CCK-8 cell proliferation assay

The effect of MAPK8IP1 overexpression and SH3GLB1 knockdown on cell proliferation was determined by Cell Counting Kit-8 (CCK-8, Dojindo, Japan) assay [52]. The T98G and LN229 tumor cells were seeded (2-4 × 10^3^ cells/well in 0.1 ml) in 96-well flat bottom plates and incubated overnight at 37°C. Then, cells were incubated with 10μl of 10% CCK-8 at 24, 48 and 72h for 1h and the absorbance was read at 450 nm in a microplate reader (IMARK). All experiments were repeated in triplicates.

### Wound healing assay and transwell invasion assay

The T98G and LN229 cells were seeded in 12-well plates and cultured until they reached confluency. Then, the cells were transfected with MAPK8IP1 plasmids or SH3GLB1 siRNAs and a scratch was created by manually scraping the cell monolayer with a 20-µl sterile pipette tip. The cells were washed twice with PBS and incubated in DMEM without FBS. The scratched area was photographed at 0 and 24h using an Axiovert 200 microscope (Carl Zeiss) and analyzed by Image proplus software. The transwell invasion assay was done in 24-well cell culture chambers using transwell inserts (Corning) with 8µm pore membranes pre-coated with matrigel (BD Bioscience). Briefly, transfected cells with DMEM were seeded in the upper chamber whereas the lower chamber was filled with 500µL DMEM with 10% FBS. After 24h, the cells in the upper surface were removed with a cotton swab, while the cells in the lower surface were fixed for 30 min in absolute ethyl alcohol, air-dried briefly and stained with 0.1% crystal violet for 10 min and counted. All experiments were performed in triplicates.

### Statistical Analysis

The bioinformatics analysis was performed with R software, version 3.0.3 (http://www.R-project.org). The experimental data was presented as mean ± SEM analyzed using SPSS version 13.0 software (Chicago, IL, USA). The Student’s t-test was used to analyze the differences between treatment and control groups. Comparisons among all groups were performed using one-way analysis of variance (ANOVA). *P*<0.05 was considered statistically significant.

## SUPPLEMENTARY MATERIALS FIGURES AND TABLES




