# Supplementary material for: Autophagy-related gene expression is an independent prognostic indicator of glioma
Source: Oncotarget. 2017 May 9;8(37):60987–1000. doi: 10.18632/oncotarget.17719 (PMC5617400; doi:10.18632/oncotarget.17719)
Supplement: Supplementary file 1 [file oncotarget-08-60987-s001.pdf]

Autophagy-related gene expression is an independent prognostic indicator of glioma

SUPPLEMENTARY MATERIALS

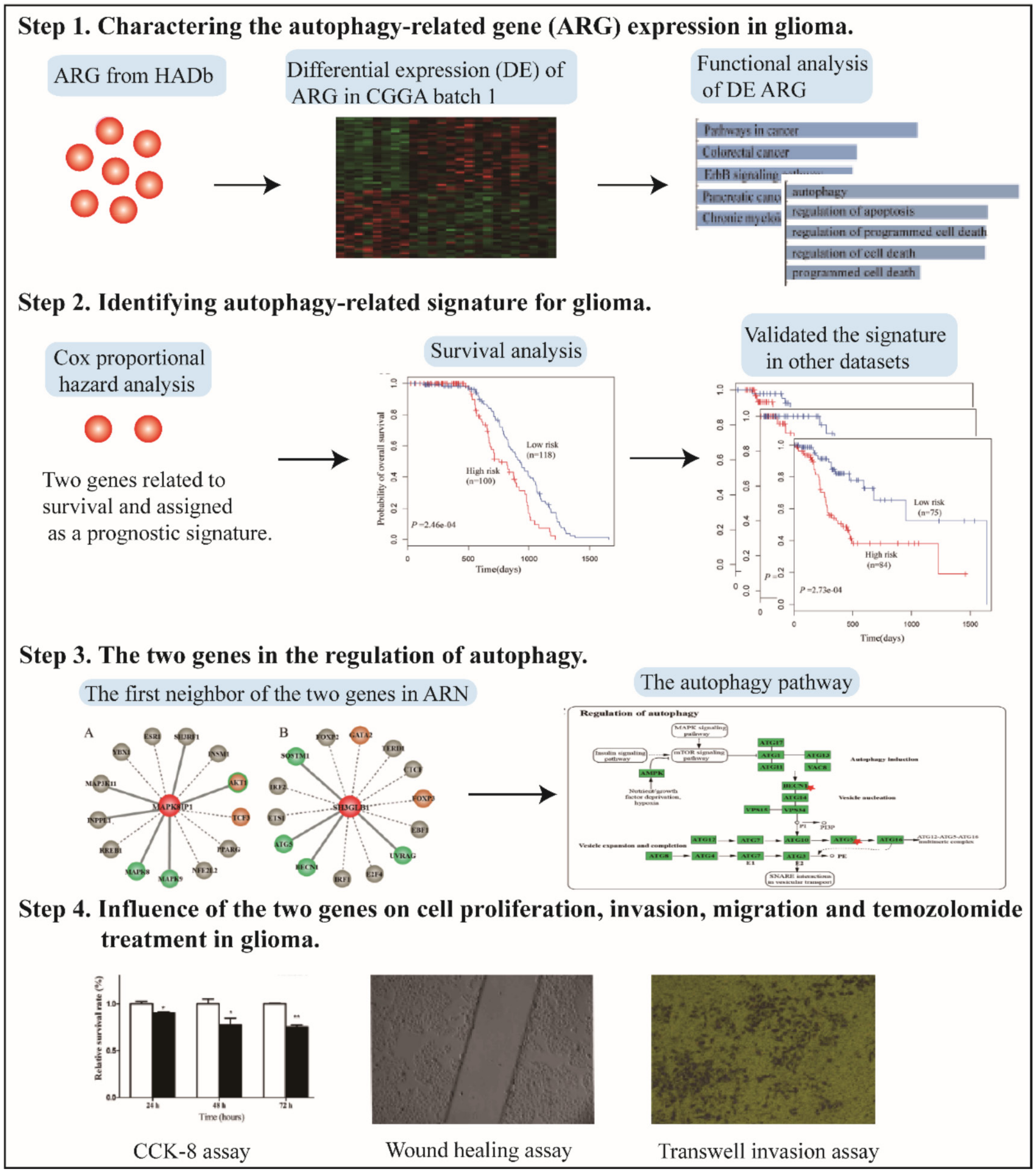

Supplementary Figure 1: The flowchart of our method. Step 1. We obtained the autophagy-related genes from the HADb and we performed functional annotation of the differentially expressed genes to characterize the autophagy-related gene expression in glioma. **Step 2.** We identified autophagy-related signature for glioma by survival analysis and found two genes were related to the overall survival of glioma patients. **Step 3.** The two genes involved in the regulation of autophagy. **Step 4.** Experiments were carried out to demonstrate the influence of the two autophagy-related genes on cell proliferation, invasion, migration and temozolomide treatment in glioma.

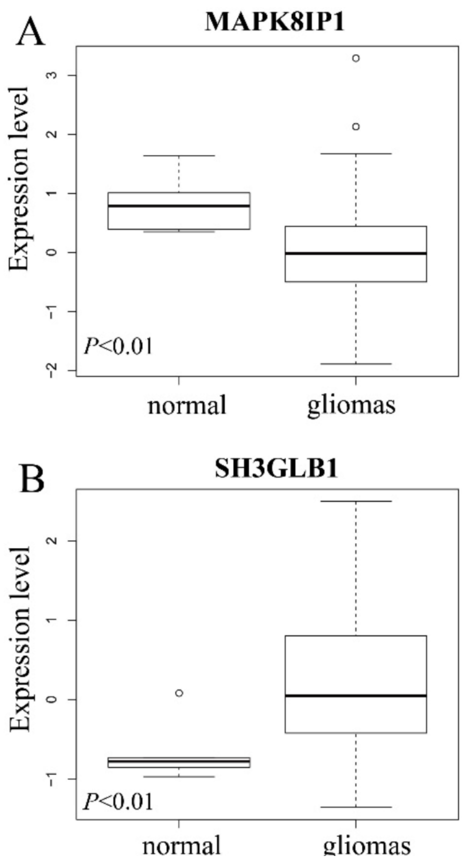

Supplementary Figure 2: mRNA expression of MAPK8IP1 and SH3GLB1 in normal and glioma tissue samples (CGGA Batch 1). MAPK8IP1 was down-regulated in glioma samples **(A)** while SH3GLB1 was up-regulated in glioma samples of CGGA Batch 1 **(B)**.

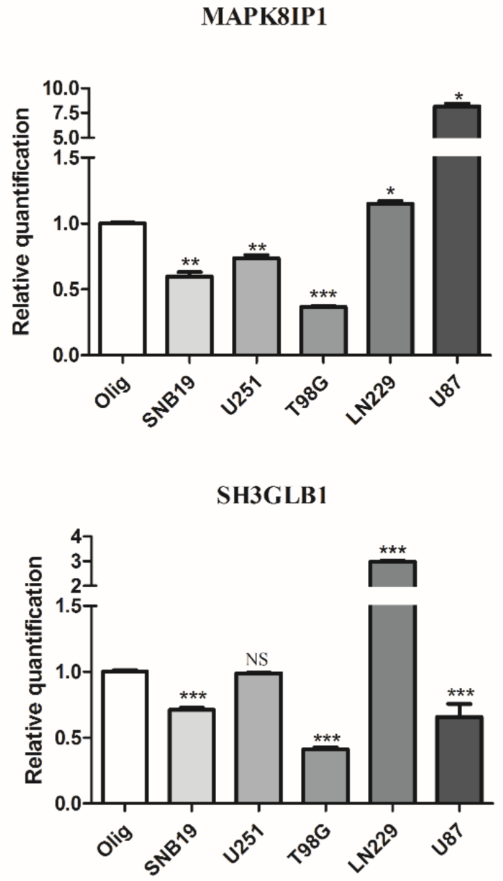

Supplementary Figure 3: mRNA expression of MAPK8IP1 and SH3GLB1 in different human glioma cell lines analyzed by qRT-PCR. (A) mRNA expression of MAPK8IP1 in five glioma cell lines and Oligodendrocyte. **(B)** mRNA expression of MAPK8IP1 in five glioma cell lines and Oligodendrocyte. Data represent mean±SEM of three replicates. $^{*}P < 0.05$; $^{**}P < 0.01$; $^{***}P < 0.001$.

Supplementary Table 1. Clinical characteristics of glioma patients according to high or low risk autophagy-related signature in the training set, the testing set and two independent cohorts.

See Supplementary File 1

Supplementary Table 2: The sequence of SH3GLB1 siRNA and Control siRNA

Product	Sequence
Si-homo-SH3GLB1-667	5′ GCUUAUGGUAAUGCCCUUATT 3′
	3′ UAAGGGCAUUACCAUAAGCTT 5′
Si-homo-SH3GLB1-972	5′ GGGAAUCAGCAGUACACAUTT 3′
	3′ AUGUGUACUGCUGAUUCCCTT 5′
Si-homo-SH3GLB1-1045	5′ GCACAGUGUUACCAGUAUATT 3′
	3′ UAUACUGGUAACACUGUGCTT 5′
Control siRNA	5′ UUCUCCGAACGUGUCACGUTT 3′
	3′ ACGUGACACGUUCGGAGAATT 5′
