# Supplementary material for: Autophagy-related gene expression is an independent prognostic indicator of glioma
Source: Oncotarget. 2017 May 9;8(37):60987–1000. doi: 10.18632/oncotarget.17719 (PMC5617400; doi:10.18632/oncotarget.17719)
Supplement: Supplementary file 2 [file oncotarget-08-60987-s002.docx]

**Supplementary Table 1. Clinical characteristics of glioma patients according to high or low risk autophagy-related signature in the training set, the testing set and two independent cohorts.**

| **Characteristic** | **Patients (high risk)** | **Patients (low risk)** | ***P* Value** |
| --- | --- | --- | --- |
| **Training set (n=218)** | n=100 | n=118 |  |
| Age (mean±SD) | 42.4±13.0 | 41.9±11.1 | 0.722**^a^** |
| Gender | | | |
| Male | 61 | 61 | 0.107**^b^** |
| Female | 39 | 57 |  |
| Stage | | | |
| II | 28 | 69 | 0.005**^b^** |
| III | 8 | 26 |  |
| IV | 64 | 23 |  |
| Histology | | | |
| Astrocytoma | 21 | 45 | 1.78×10^-6^**^b^** |
| Glioblastoma | 64 | 23 |  |
| Oligodendrocytoma | 4 | 24 |  |
| Others | 11 | 26 |  |
| **Testing set (n=84)** | n=38 | n=46 |  |
| Age (mean±SD) | 49.7±10.2 | 41.3±10.8 | 0.0005**^a^** |
| Gender | | | |
| Male | 26 | 30 | 0.47**^b^** |
| Female | 12 | 16 |  |
| Stage | | | |
| II | 1 | 27 | 0.134**^b^** |
| III | 6 | 11 |  |
| IV | 31 | 8 |  |
| Histology | | | |
| Astrocytoma | 1 | 9 | 9.929×10^-5^**^b^** |
| Glioblastoma | 31 | 8 |  |
| Oligodendrocytoma | 0 | 10 |  |
| Others | 6 | 19 |  |
| **Independent cohort (GSE4412, n=85)** | n=36 | n=49 |  |
| Age (mean±SD) | 46±15.5 | 43.2±15.5 | 0.41**^a^** |
| Gender | | | |
| Male | 16 | 16 | 0.189**^b^** |
| Female | 20 | 33 |  |
| Stage |  |  |  |
| III | 3 | 23 | 1.0**^b^** |
| IV | 33 | 26 |  |
| Histology | | | |
| Astrocytoma | 1 | 7 | 2.526×10^-6^**^b^** |
| Glioblastoma | 33 | 26 |  |
| Oligodendrocytoma | 0 | 11 |  |
| Others | 2 | 5 |  |
| **Independent cohort (TCGA, n=159)** | 84 | 75 |  |
| Age (mean±SD) | 61.3±13.6 | 57.8±13.6 | 0.104**^a^** |
| Gender | | | |
| Male | 51 | 53 | 0.931**^b^** |
| Female | 33 | 22 |  |

**^a^ Student’s t test.**

**^b^ Fisher’s exact test.**
